# Pharmacokinetics of Nitrate and Nitrite Following Beetroot Juice Drink Consumption

**DOI:** 10.3390/nu13020281

**Published:** 2021-01-20

**Authors:** Emily Margaret Jakubcik, Kay Rutherfurd-Markwick, Marsanne Chabert, Marie Wong, Ajmol Ali

**Affiliations:** 1 School of Sport, Exercise and Nutrition, Massey University, Auckland 0745, New Zealand; emilyjakubcik@outlook.com; 2 School of Health Sciences, Massey University, Auckland 0745, New Zealand; K.J.Rutherfurd@massey.ac.nz; 3 Centre for Metabolic Health Research, Massey University, Auckland 0745, New Zealand; 4 Institut Polytechnique de Bordeaux, 33300 Bordeaux, France; marsannechabert@orange.fr; 5 School of Food and Advanced Technology, Massey University, Auckland 0745, New Zealand; M.Wong@massey.ac.nz

**Keywords:** nitric oxide, cardiovascular disease, cardioprotective, blood pressure

## Abstract

Background: Nitrate (NO_3_^−^)-rich beetroot (BR) juice supplementation has been shown to improve cardiovascular function via reduction to nitrite (NO_2_^−^) and then to the bioactive molecule nitric oxide (NO). However, limited research exists for the role of inorganic NO_2_^−^ that is contained naturally within BR. Objective: As BR juice can naturally contain both NO_3_^−^ and NO_2_^−^ the objective of this study was to evaluate the individual effects of NO_3_^−^ and NO_2_^−^ consumed from BR on plasma [NO_3_^−^]/[NO_2_^−^] and their subsequent effects on various cardiovascular measures. Design: In four separate treatments, 11 healthy adults consumed 250 mL of BR containing one of the following: (i) high NO_3_^−^, low NO_2_^−^ (HL; 572 mg NO_3_^−^, 32 mg NO_2_^−^); (ii) medium NO_3_^−^, medium NO_2_^−^ (MM; 280 mg NO_3_^−^, 237 mg NO_2_^−^); (iii) low NO_3_^−^, medium NO_2_^−^ (LM; 43 mg NO_3_^−^, 262 mg NO_2_^−^); (iv) placebo (PL; low NO_3_^−^, low NO_2_^−^: 8 mg NO_3_^−^, 5.8 mg NO_2_^−^). Plasma [NO_3_^−^]/[NO_2_^−^], blood pressure, heart rate, mean arterial pressure (MAP), cardiac output and stroke volume were measured at baseline and every hour or second hour for 6 h post-BR consumption. Outcomes: Ingestion of the HL and MM BR increased plasma [NO_2_^−^] and [NO_3_^−^] after 2 h, with both remaining elevated after 6 h (*p* < 0.05). LM increased plasma [NO_3_^−^] (*p* < 0.05) but did not increase plasma [NO_2_^−^] compared to PL (*p* = 0.177). MAP was lower following the consumption of HL at 4 h and LM at 6 h (*p* < 0.05). Conclusion: Inorganic NO_3_^−^ consumption is the critical factor in elevating plasma [NO_3_^−^] and [NO_2_^−^]; however, both NO_2_^−^ and NO_3_^−^ show potential to reduce MAP. The known reduction of systolic blood pressure (SBP)/diastolic blood pressure (DBP) following NO_3_^−^ supplementation was not observed, making it unclear if NO_2_^−^ contributes to a reduction in SBP/DBP alongside NO_3_^−^.

## 1. Introduction

Cardiovascular disease (CVD) places a significant burden on the health system, accounting for approximately one-third of all deaths [1]. A major risk factor for the development of CVD is increased blood pressure (BP) or hypertension [2,3] which is often the target for CVD-based interventions. Epidemiological evidence suggests a diet rich in fruit and vegetables reduces BP and the subsequent risk of CVD [4,5,6,7]. This effect has been previously attributed to the abundance of antioxidants and vitamins which are present in fruit and vegetables [4,7]. However, other studies failed to see any cardioprotective effect of antioxidants and vitamins [8,9,10], thus prompting researchers to investigate potential cardioprotective effects of other compounds present in fruits and vegetables. While it is likely that the cardioprotective effect observed with fruit and vegetable intake is multifactorial, the consumption of high nitrate (NO_3_^−^) vegetables [11,12] has been shown to lead to the greatest reduction in BP compared to other vegetables [13,14]. As a result, there has been increased interest in the potential of NO_3_^−^ derived from fruit and vegetables to lead to a reduction in BP [13].

Beetroot juice (BR) is used as a supplement because of its high inorganic nitrate (NO_3_^−^) content, a compound found naturally in vegetables and in processed meats, where it is used as a preservative [15]. Beetroot juice contains varying amounts of both inorganic NO_3_^−^ and inorganic nitrite [16,17,18] which in the human body can reduce to the bioactive molecule nitric oxide (NO) through an exogenous pathway [19]. This exogenous pathway begins with the absorption of NO_3_^−^ in the gastrointestinal tract, which is then distributed to various locations, including the mouth [16]. In the mouth NO_3_^−^ is reduced to NO_2_^−^ by anaerobic bacteria [20]. NO_2_^−^ is then swallowed and re-enters the gastrointestinal tract; when it reaches the stomach, the acidic environment reduces NO_2_^−^ to NO [21]. NO enters the circulation and is then able to lower BP by relaxing and dilating the endothelium [22,23,24].

Consistent with the exogenous pathway, multiple studies have reported a reduction of systolic BP (SBP; 5–22 mmHg) and diastolic BP (DBP; 2.4–18.3 mmHg) following supplementation with BR [20,25,26,27,28,29]. This effect has been shown to occur in a dose-dependent manner, with a larger dose of NO_3_^−^ reducing BP to a greater extent than a smaller dose [25,29,30]. However, some studies have shown no change in either SBP or DBP following BR consumption [31,32]. Although the exact reason for the contradictory data is unclear, it may relate to dosage level—one of these studies [31] used a low NO_3_^−^ dose (119 mg; 1.4 mmol/day) which was likely insufficient to result in a reduction in BP.

Interestingly, several studies support NO_2_^−^ having a cardioprotective effect [33,34,35]. Specifically, in humans the oral consumption of NO_2_^−^ via NaNO_2_^−^ elicits an elevated plasma NO_2_^−^ [36], which correlates with a reduction in BP [37,38,39]. When specifically investigating the cardioprotective effect of NaNO_2_^−^ capsules (80 mg NO_2_^−^ or 160 mg NO_2_^−^), an improvement in brachial artery flow was seen but, interestingly, no change in BP [40]. Studies in mice have shown that NO_2_^−^ infusion led to a reduction in mean arterial pressure [41,42] and reversed endothelial dysfunction [43]. The cardioprotective effects of NO_2_^−^ can be explained via the exogenous NO pathway, and the ability for NO_2_^−^ to also produce the bioactive molecule NO [21,40]. While there is potential for NO_2_^−^ to reduce BP via the exogenous NO pathway, to date there has been no consideration as to whether the NO_2_^−^ in BR contributes to the observed reductions in BP.

Therefore, we determined the impact of different doses of NO_3_^−^ and NO_2_^−^ from BR on plasma levels of NO_3_^−^ and NO_2_^−^ and examined the effect of these different doses on cardiovascular measures. In addition, correlations between the plasma NO_3_^−^ and NO_2_^−^ levels and physiological outcomes were investigated. 

## 2. Methods and Materials

### 2.1. Participants

Eleven healthy adults (18–50 years old) volunteered for this study (5 M; 6 F). All participants completed a health-screening questionnaire to determine eligibility. Inclusion criteria included healthy men and pre-menopausal women aged 18–50 years who were proficient in English and could attend the laboratory on multiple occasions. Those who regularly consumed NO_3_^−^-based dietary supplements, or were unable to participate in blood collection, or had a beetroot allergy, or were pregnant or had known health issues, e.g., hypertension or cardiovascular disease, were excluded. Prior to their involvement, the study was explained to all participants and each was provided with an information sheet. Participants were fully informed of any risks associated with the experiments before giving their informed written consent to participate in the investigation. The study was approved by the Massey University Human Ethics Committee (SOA 18/35).

### 2.2. Study Design and Procedures

Participants were required to visit the laboratory (18–20 °C, 40–60% relative humidity) on five occasions. During the first visit they were familiarised with the testing procedures and equipment, including an ultrasonic cardiac output monitor (USCOM1A; Uscom Ltd.; Sydney, Australia) and automated sphygmomanometer (deluxe HEM-7310; OMRON Healthcare CO. Ltd.; Kyoto, Japan). Height was measured using a stadiometer (Seca portable stadiometer, Amtech, New Zealand) and body mass using scales accurate to 0.1 kg (A & D Weighing, HV-200KGL, Adelaide, Australia).

For 24 h prior to visit 2, participants recorded their food and fluid intake and were asked to replicate these diets for each 24-h period prior to the remaining visits (3 to 5). Participants were instructed to arrive at the laboratory in a fasted state, and to have refrained from caffeine ingestion, strenuous exercise and alcohol for 24 h pre-trial. Each trial commenced in the morning at approximately the same time of day (9 a.m. +/− 1 h). 

Each participant was randomly allocated the order which the supplemented beverages would be consumed in a double-blinded, randomized crossover design. Visits 2 to 5 were the experimental trials and each trial was separated by a one-week washout period to allow for complete normalization of nitrate levels in the body [44,45,46]. The drink was consumed within a 10-min period alongside a standardised, isocaloric breakfast with a macronutrient distribution of 15 g protein, 30 g carbohydrate and 10 g fat. Three hours post-supplement intake the participants received a standardised, isocaloric lunch with a macronutrient distribution of 25 g protein, 45 g carbohydrate and 16 g fat. 

**Figure 1:**
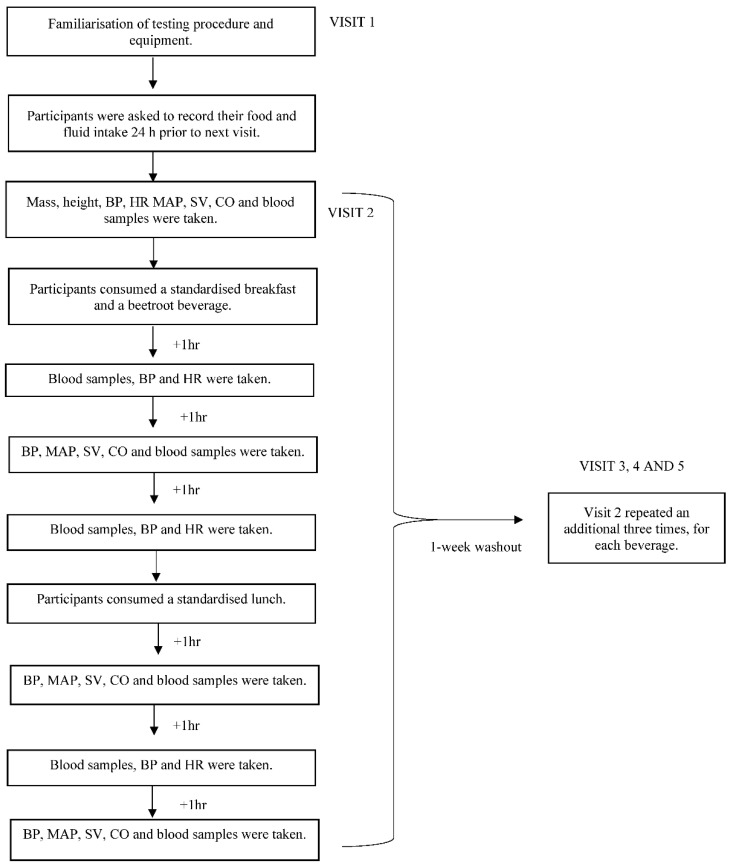
Protocol of the experimental trials. BP, blood pressure; HR, heart rate; SV, stroke volume; MAP, mean arterial pressure; CO, cardiac output.

On experimental trial days, baseline measures of blood pressure and a resting blood sample were taken on arrival and prior to supplementation. The allocated drink and breakfast were consumed and the timer started immediately after the drink was finished. Blood samples, BP and heart rate (HR) were measured each hour and other hemodynamic measurements (stroke volume (SV), mean arterial pressure (MAP) and cardiac output (CO)) were carried out via the USCOM every second hour (0 h, +2 h, +4 h, +6 h). Participants remained in the laboratory for the duration of the trial and were able to complete office work between sampling periods. The study protocol is illustrated in Figure 1. 

### 2.3. Intervention

For each of the experimental trials, participants consumed 250 mL of one of the four juices containing different levels of nitrate and nitrite (Table 1). Each drink was similar in both appearance and taste and was not perceived as significantly different (*p* > 0.05) when presented to a consumer sensory panel [47]. The high and medium doses of NO_3_^−^ (572 mg and 280 mg, respectively) were chosen as these levels have been shown to be sufficient to elicit cardioprotective effects [29]. The chosen nitrite dose was the variable being tested—due to the low concentration of naturally occurring nitrite we were unable to produce a beverage containing >500 mg nitrite. 

Freshly prepared BR was blended with apple juice and water, and the flavour adjusted with a natural beetroot powder (low in NO_3_^−^) to produce two standardized drinks of a constant soluble solids concentration (11 °Brix). These drinks were each used as they were and further as the base drink for one other formulation. The drinks were pasteurized and poured into 250 mL plastic bottles, stored frozen and then thawed immediately prior to use. The placebo (PL) was made to a standardized low nitrate (8 mg/250 mL) and low nitrite (5.8 mg/250 mL) concentration which also formed the base of the high NO_3_^−^, low NO_2_^−^ (HL) beverage. The low nitrate, medium nitrite (LM) beverage was standardized to contain 43 mg nitrate and 262 mg nitrite per 250 mL. This drink was also used as the base formulation for the medium nitrate and medium nitrite beverage (MM). 

The medium nitrate and medium nitrite beverage (MM) was prepared immediately prior to consumption by replacing 30 mL of the LM beverage with 30 mL of a commercial BR with known NO_3_^−^ and NO_2_^−^ content (determined by high-performance liquid chromatography (HPLC)). This produced a 250 mL beverage of 11 ° Brix containing standardized medium nitrate (280 mg/250 mL) and medium nitrite (237 mg/250 mL). Similarly, to prepare the HL solution, 70 mL of the placebo drink was replaced with 70 mL of the commercial BR immediately prior to consumption to produce a 250 mL beverage of 11° Brix containing standardized high nitrate (572 mg/250 mL) and low nitrite (32 mg/250 mL). The final concentrations of both nitrate and nitrite within the drinks were determined using high-performance liquid chromatography.

### 2.4. Blood Measurements 

Venous blood samples (6 mL) were taken via a cannula (where possible) or by venepuncture within the antecubital area and collected into heparinized tubes. Following cannula blood sampling, 5 mL of a noncoagulant saline solution was then injected through the cannula to prevent clotting and blockage of the line. Samples were mixed and immediately centrifuged (MF-50 Hanil Science Industrial, Gimposi, Korea) at 1300× *g* for 10 min, and the collected plasma was aliquoted and stored at −80 °C for later HPLC analysis of NO_3_^−^ and NO_2_^−^. 

### 2.5. HPLC Analysis

The quantification of NO_3_^−^ and NO_2_^−^ in beetroot juice was based on previously described methods [48,49]. Briefly, samples were centrifuged, filtered (0.45 µm), diluted and separated on a Gracesmart C-18 column (4.6 × 250 mm; 5 µm), using an isocratic mobile phase of 0.01 M Octylammonium orthophosphate (pH 3–3.5) at a flow rate of 0.8 mL/min (20 °C). Detection occurred at UV wavelengths of 193 nm for NO_2_^−^ and 213 nm for NO_3_^−^. Quantification was achieved with external standard Na NO_3_^−^ and Na NO_2_^−^ solutions.

The quantification of NO_2_^−^ in plasma was based on the method described by Li et al., [50]. The plasma samples were filtered using 10 kD cut-off filters (Amicon Ultra 2 10 KDa 2 mL Thermo LPN 00288739 UFC201024) which had each been washed four times with water. The determination of NO_2_^−^ occurs through its derivatisation with 2,3-diaminonaphthalene (DAN) to yield the highly fluorescent 2,3-naphthotriazole (NAT). Briefly, NAT was separated at a flow rate of 1.3 mL/min (20 °C) on a Waters Spherisorb S5C8 column (4.6 × 250 mm; 5 µm) using a mobile phase of 15 mM sodium phosphate (pH 7.5) containing 50% methanol (0–5 min), sequentially followed by 100% Milli-Q water (5.1–7 min), 100% methanol (7.1–10 min) and 100% Milli-Q water (10.1–12 min) before returning to the initial buffer (12.1–17 min). Fluorescence was monitored with excitation at 375 nm and emission at 415 nm. 

This same filtration process was utilized for plasma NO_3_^−^ analysis. Following the filtration, the method previously described to quantify the NO_3_^−^ within juice was used to quantify the NO_3_^−^ within plasma following a suitable dilution. 

### 2.6. Cardiovascular Measurements 

Blood pressure and HR were measured via an automated sphygmomanometer (mean value of three measurements taken from the left arm [51]. The USCOM was used to measure velocity time integral (VIT), HR, SBP and DBP. Utilizing these measurements and various algorithms, the mean arterial pressure, stroke volume and cardiac output were obtained. 

### 2.7. Statistical Analysis

Data were checked for normality using the Shapiro–Wilk test and variance using Levene’s test. Data were expressed as mean ± SD and all analyses were completed on raw data and delta-changed data to eliminate the effect of baseline differences. A *p*-value < 0.05 was considered to be statistically significant. For groups showing statistical differences, an effect size was calculated, with 0.2 being considered a ”small” effect size, 0.5 being considered a ”medium” effect size and 0.8 a “large” effect size [52]. All statistical analyses were completed using IBM SPSS package version 22 (IBM Corporation, Chicago, IL, USA).

Any differences between individual baseline values for all variables for both different drinks and different trial numbers were determined using one-way ANOVA. The main effect of drink, the main effect of time and the interaction effect of drink and time were determined for each variable using a repeated measures two-way ANOVA. To ensure accuracy, both the measured values (absolute value) and the change from baseline (delta-change) were analyzed. Additionally, to exclude the effect of lunch, analysis of each variable was completed taking into account pre-lunch time points (0 h to 3 h) along with the analysis on all time points (0 h to 6 h) for both forms of data. To limit confounders, this was repeated for the main effect of trial, the main effect of time and the interaction effect of trial and time. When a significant interaction was found, Holm–Bonferroni post-hoc tests were used to find out where differences lay. 

Pearson’s correlation was used to investigate relationships between plasma [NO_2_^−^] and [NO_3_^−^] and cardiovascular measures; where 0.2 inferred a weak correlation, 0.5 inferred a moderate correlation and 0.8 inferred a strong correlation [53].

## 3. Results 

### 3.1. Participants

All eleven participants completed the BP, HR and USCOM measures. Of the eleven, only eight participants completed all blood samples due to difficulties during the blood collection process. Participant characteristics are reported in Table 2. 

#### 3.1.1. Plasma NO_2_^−^

**Figure 2:**
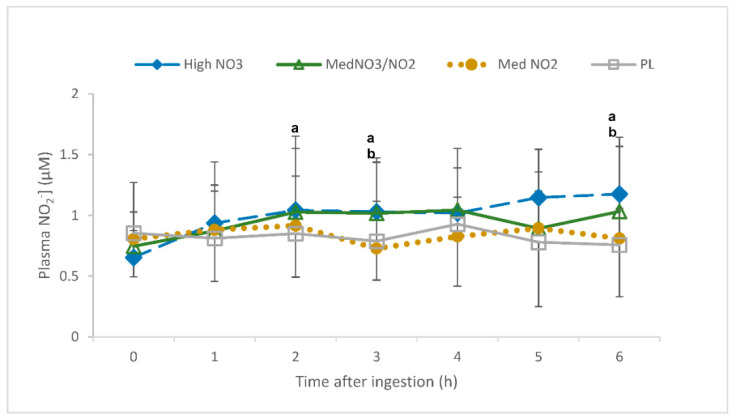
Plasma nitrite concentration ([NO_2_^−^]) (uM) over a 6-h period following the ingestion of placebo (PL); high nitrate, low nitrite (HL); low nitrate, medium nitrite (LM); and medium nitrate and medium nitrite (MM) beverages. ^a^ Significant difference between HL and PL (*p* < 0.05). ^b^ Significant difference between HL and LM (*p* < 0.05).

There was an interaction of drink and time for plasma [NO_2_^−^] (*p* < 0.001). Specifically, plasma [NO_2_^−^] was elevated in the high NO_3_^−^ trial relative to the high NO_2_^−^ (3 h and 6 h post-consumption) and PL (2 h and 6 h post-consumption; *p* < 0.05; Figure 2). PL and high NO_2_^−^ consumption showed no increases in plasma [NO_2_^−^] at any time point (*p* > 0.05). The delta change values also showed an elevation in plasma [NO_2_^−^] following consumption of HL relative to PL and LM (3 h and 6 h; *p* < 0.05). Plasma [NO_2_^−^] had a higher increase in the MM trial relative to PL (3 h and 6 h) and LM (3 h; *p* < 0.05); however, it was still lower than HL at 6 h (*p* < 0.05) but not at 3 h (*p* = 0.16). 

#### 3.1.2. Plasma NO_3_^−^

**Figure 3:**
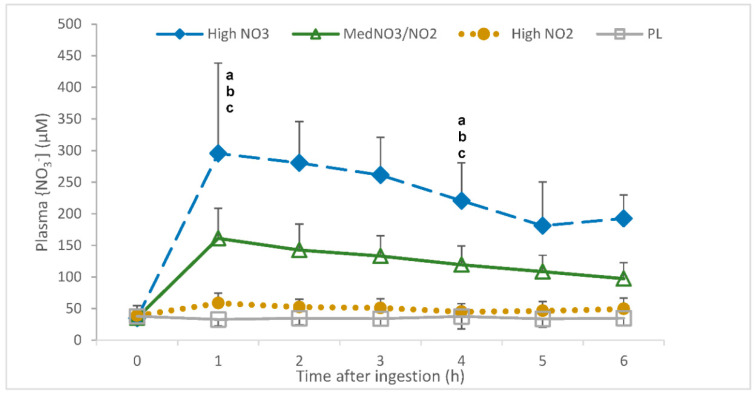
Plasma nitrate concentration ([NO_3_^−^]) (uM) over a 6-h period following the ingestion of placebo (PL); high nitrate, low nitrite (HL); low nitrate, medium nitrite (LM); and medium nitrate and medium nitrite (MM) beverages. ^a^ Significant difference between HL and PL (*p* < 0.05). ^b^ Significant difference between HL and LM (*p* < 0.05). ^c^ Significant difference between HL and MM (*p* < 0.05).

There was an interaction of drink and time for plasma [NO_3_^−^] (*p* < 0.001). Specifically, plasma [NO_3_^−^] was elevated in the HL, MM and LM trials relative to PL (2 h and 6 h post-consumption; *p* < 0.01; Figure 3). In all trials, time was shown to increase plasma [NO_3_^−^], rising from 1 h (*p* < 0.001) and plateauing at 5 h (5 h vs 6 h *p* = 0.80). The delta change values showed that the highest increase in plasma [NO_3_^−^] occurred following the consumption of HL (*p* < 0.001), with the second highest increase occurring following the consumption of MM (*p* < 0.001). 

### 3.2. Blood Pressure (BP)

There was no interaction of drink and time for SBP (*p* = 0.325). SBP was reduced in all trials (2 h, 3 h, 5 h and 6 h post-consumption; *p* < 0.001), with no difference occurring between trials (*p* = 0.783). These findings were consistent when analysing delta change and pre-lunch data. 

There was a trend for an interaction of drink and time for absolute data and change in diastolic blood pressure (*p* = 0.059; *p* = 0.058); this trend was not present pre-lunch (*p* > 0.05). There was no difference in DBP between trials (*p* = 0.692) and no effect of time on DBP (*p* = 0.124). 

### 3.3. Heart Rate

There was no interaction of drink and time for HR in absolute or delta change data (*p* > 0.05). However, there was an interaction of drink and time for pre-lunch HR. Specifically, HR was reduced in the PL trials relative to MM (3 h post-consumption, *p* < 0.05).

There was a main effect of time for HR (*p* < 0.05). Specifically, in all trials HR was reduced, 1 h, 4 h, 5 h and 6 h post-consumption (*p* < 0.05), with the greatest decrease in HR occurring at 3 h (*p* < 0.001).

### 3.4. USCOM Measures

There was no main effect of time or treatment or interaction of drink and time for CO or SV (all *p* > 0.05). These findings were consistent when analysing delta change and pre-lunch data.

**Figure 4:**
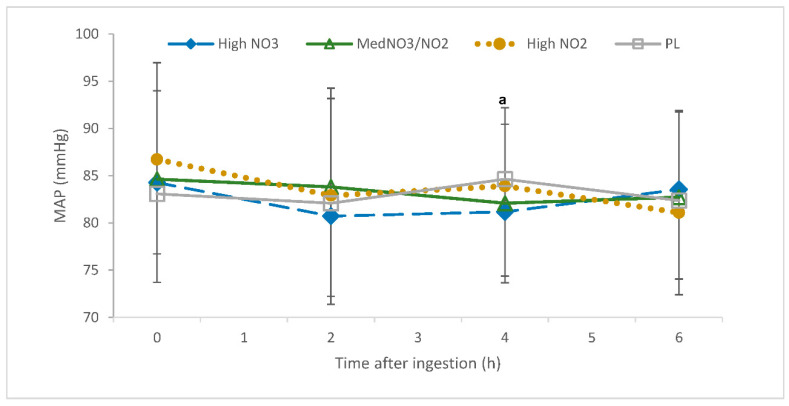
Mean arterial pressure (mmHg) over a 6-h period following the ingestion of placebo (PL); high nitrate, low nitrite (HL); low nitrate, medium nitrite (LM); and medium nitrate and medium nitrite (MM) beverages. ^a^ Significant difference between HL and LM *p* < 0.05).

**Figure 5:**
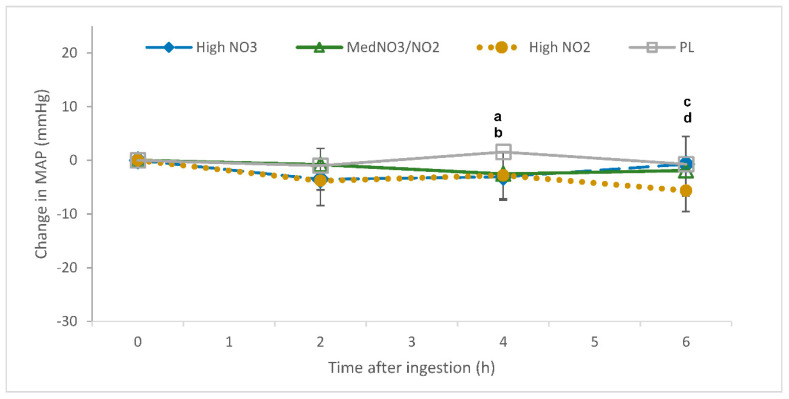
Change in mean arterial pressure (mmHg) over a 6-h period following the ingestion of placebo (PL); high nitrate, low nitrite (HL); low nitrate, medium nitrite (LM); and medium nitrate and medium nitrite (MM) beverages. ^a^ Significant difference between HL and PL (*p* < 0.05). ^b^ Significant difference between MM and PL (*p* < 0.05). ^c^ Significant difference between HL and LM (*p* < 0.05). ^d^ Significant difference between LM and PL (*p* < 0.05).

There was an interaction of drink and time for mean arterial pressure (*p* = 0.013). MAP was reduced in the HL trial relative to LM (4 h post-consumption; *p* < 0.05; Figure 4). The delta change values also showed an interaction of drink and time for MAP, which was reduced in the HL and MM trials relative to PL (4 h post-consumption; *p* < 0.05; Figure 5) and in the LM group relative to HL and PL (6 h post-consumption; *p* < 0.05; Figure 5).

### 3.5. Correlations

Plasma [NO_3_^−^] showed a weak correlation with SBP at 6 h (r = 0.428; *p* < 0.006). Additionally, a weak negative correlation was also seen with the delta change data, where plasma [NO_3_^−^] correlated with DBP at 4 h (r = −0.364; *p* = 0.021). 

## 4. Discussion

This study examined the effect of acute supplementation with beetroot juice rich in either NO_3_^−^ or NO_2_^−^, or a combination of both, on plasma [NO_3_^−^], plasma [NO_2_^−^] and cardiovascular responses in normotensive adults. The main findings of this study demonstrate that: (1) NO_3_^−^ consumption is the critical factor in elevating both plasma [NO_3_^−^] and [NO_2_^−^]; (2) NO_3_^−^ ingestion reduces MAP 4 h post-consumption while NO_2_^−^ reduces MAP 6 h post-consumption; (3) an increase in plasma [NO_3_^−^] leads to decreased DBP after 4 h.

### 4.1. Plasma [NO_2_^−^] and [NO_3_^−^] 

This is the first study to directly evaluate the effects of NO_2_^−^ present in BR on plasma [NO_2_^−^] and [NO_3_^−^]. Both HL and MM increased plasma [NO_3_^−^] and [NO_2_^−^] to a greater extent than LM and PL, implying that NO_3_^−^ consumption is the critical factor for the rise in plasma [NO_3_^−^] and [NO_2_^−^]. Furthermore, HL caused a higher rise in both plasma [NO_3_^−^] and [NO_2_^−^] than MM, a change which is proportional to the [NO_3_^−^] within the drink. Both HL and MM drinks contained similar combined quantities of NO_3_^−^ and NO_2_^−^ (604 mg and 517 mg respectively), but different quantities of NO_3_^−^ (572 mg; 280 mg respectively) and NO_2_^−^ (32 mg; 237 mg respectively), leading to the conclusion that there is little effect of NO_2_^−^ on plasma [NO_3_^−^] or [NO_2_^−^].

Following the consumption of HL, peak plasma [NO_3_^−^] occurred after 1 h, and peak plasma [NO_2_^−^] occurred after 2 h. These findings are consistent with studies in the literature which showed NO_3_^−^-rich BR led to peak plasma [NO_3_^−^] 1–1.5 h post-ingestion [54] and peak plasma [NO_2_^−^] 2.5–3 h post-ingestion [55]. Furthermore, the current study showed that following the consumption of LM, peak plasma [NO_3_^−^] occurred 1 h post-ingestion but with no increase in plasma [NO_2_^−^]. This result is inconsistent with previous studies, where the ingestion of NaNO_2_^−^ capsules increased both plasma [NO_3_^−^] and [NO_2_^−^], leading to the conclusion that NO_2_^−^ was 95–98% bioavailable (compared to the same IV dose) [36]. Although the source of supplement was different between our study and Hunault et al.’s [36], there were comparable doses of NO_2_^−^ (193–253 mg vs. 262 mg). Although specific research comparing the effect of NO_2_^−^ supplementation between BR and capsules does not exist, it has been suggested that synthetic nutrients delivered in capsules may not be used by the body in the same way as their natural counterparts [7]. 

Previous literature suggests that a reduction in BP following BR consumption (400–800 mg NO_3_^−^) is reliant on a rise in plasma [NO_2_^−^] (0.5–0.9 μmol/L) [37,38,39]. Our findings show for the first time that BR high in NO_3_^−^ but low in NO_2_^−^ (HL) led to the greatest rise in plasma [NO_2_^−^], and therefore NO_3_^−^ consumption may be the critical factor in producing a reduction in BP following BR supplementation. Furthermore, when BR is stored at room temperature, NO_3_^−^ begins to convert to NO_2_^−^ [17]; hence when BR is left at room temperature for a period of time, it may not lead to the same increase in plasma [NO_2_^−^] and therefore may have a reduced ability to lower BP. The current study did not show a correlation between BP and plasma [NO_2_^−^] despite similar NO_3_^−^ ingestion to past studies [26,29]. 

### 4.2. Blood Pressure

#### 4.2.1. Systolic and Diastolic Blood Pressure

SBP and DBP decreased following the consumption of all drinks, including PL, suggesting that participants were becoming more relaxed over time. Interestingly, the reduction of SBP was not different between trials, leading to the conclusion that neither NO_3_^−^ or NO_2_^−^ had an influence on SBP. Similarly, participants’ DBP did not differ after consuming the different drinks; however, there was a trend for HL to reduce DBP more than the other drinks. This may be secondary to the young, healthy participants who volunteered for this study. 

Very few studies show no reduction in either SBP or DBP following NO_3_^−^ supplementation [31,32]. More commonly, it is concluded that NO_3_^−^ supplementation (comparable doses to the present study) elicits a reduction in SBP [20,25,26,27,28,29,30,44,56] and DBP [25,27,28,29,30,57]. In studies where an effect was not observed, the presence of confounding factors preventing NO_3_^−^ from reducing BP has been suggested. For example, Floyd et al. [32] suggested that an elevation in plasma [glucose] and [insulin] can prevent a reduction in BP in a younger population (27 ± 6.5 years) following potassium NO_3_^−^ supplementation (2.4 g; 24 mmol NO_3_^−^). In the current study, 15 g of carbohydrate was consumed alongside the supplement, with a further 30 g consumed at lunch (+4 h) which may have prevented a reduction in BP due to the rise in [glucose] and [insulin]. However, the low baseline SBP of 113 mmHg in the participants in Floyd et al. [32] and in the current study (111 mmHg) may better explain the lack of effect. A metaregression, including 19 eligible randomised clinical trials investigating the effects of BR supplementation (316–860 mg NO_3_^−^/day) over 2–56 d, showed that a larger decrease in SBP occurred in participants with a higher baseline SBP measure [58]. Most previous studies which have shown BR consumption to have an effect on SBP had participants with baseline SBP measures ranging between 127–149 mmHg [20,27,28,29,30,31].

#### 4.2.2. Cardiovascular Functions

The current study showed no change in HR, SV or CO following NO_3_^−^-rich BR consumption, which is consistent with previous literature [27,59]. The present study has further shown that consumption of NO_2_^−^-rich BR also had no effect on these variables. The cardioprotective effects observed in previous literature are likely mediated by the relaxation and dilation of the endothelium associated with NO production [21,60]. Additionally, the endogenous formation of NO, via l-arginine supplementation, has shown to limit noradrenaline production, depressing sympathetic activity and increasing the parasympathetic tone [61]. A main characteristic of the sympathetic drive is increased HR, CO and SV [62], implying that endogenous NO has the ability to reduce HR, CO and SV [63]. Exogenous NO_3_^−^ has not been investigated, but the current study, along with previous literature, suggest that this does not have the same effect on noradrenaline and hence does not have the same effect on HR, CO and SV that endogenous NO_3_^−^ does. 

It has been concluded that NO_3_^−^-rich supplementation reduces BP, more significantly in those with a higher baseline BP. As the current study failed to show any BP-lowering effect associated with NO_3_^−^, it is unlikely that any effect of NO_2_^−^ on BP would be shown. Further research into the potential effects of NO_2_^−^ on SBP and DBP is required in a population with a higher baseline SBP. 

#### 4.2.3. Mean Arterial Pressure

NO_3_^−^, independent of dose (HL and MM), reduced MAP after 4 h, and NO_2_^−^ (LM) reduced MAP after 6 h. Due to the direct conversion of NO_2_^−^ to NO in the body, high NO_2_^−^ was proposed to provide an early onset of cardioprotective effects compared to high NO_3_^−^ [21,64]. Previous literature has shown a decrease in MAP (−8.2 ± 7.6 mmHg; −7 ± 1 mm Hg) following NO_3_^−^-rich BR consumption (300 mg; 450 mg) [65,66], specifically occurring 4 h post-supplementation [66], which is consistent with the current study. However, the delayed effect of NO_2_^−^ on MAP in comparison to NO_3_^−^ cannot be explained by the current literature and is not consistent with the exogenous NO pathway. Further studies are required to ensure that this is an accurate and replicable outcome.

Further research investigating the pharmacokinetics of the NO_2_^−^ present in BR, and the subsequent correlations of both plasma [NO_3_^−^] and [NO_2_^−^], is warranted. The inconsistent findings of SBP and DBP associated with NO_3_^−^-rich BR in the current study indicate a need for further research to confirm the effect of NO_2_^−^ present in BR. Future studies should include participants with a higher baseline SBP as this has been associated with a larger reduction in SBP.

## 5. Conclusions

Plasma [NO_3_^−^] increased in a dose-dependent manner following the ingestion of combined NO_2_^−^ and NO_3_^−^, while plasma [NO_2_^−^] increased following the ingestion of NO_3_^−^ only. There was a significantly higher increase of plasma [NO_3_^−^] following HL supplementation compared to MM supplementation. There was no effect of NO_3_^−^ or NO_2_^−^ ingestion on SBP, DBP, SV or CO compared to PL. MAP decreased following the consumption of HL, MM and LM; however, in comparison LM showed its effect 2 h later. Collectively, these results indicate that consumption of NO_3_^−^ is the critical factor in elevating plasma [NO_3_^−^] and [NO_2_^−^], although the consumption of either NO_3_^−^ or NO_2_^−^ can reduce MAP. Further research is required in a population with a higher baseline BP so any effects of NO_2_^−^ consumption on SBP/DBP can be observed.

## Figures and Tables

**Table 1:** Nitrate and nitrite content of the drinks (per 250 mL) (as determined by high-performance liquid chromatography (HPLC)).

Drink	Nitrate mg	Nitrite mg	Nitrate mmol	Nitrite mmol
High NO_3_^−^, low NO_2_^−^ (HL)	572	32	6.72	0.46
Medium NO_3_^−^, medium NO_2_^−^ (MM)	280	237	3.29	3.43
Low NO_3_^−^, medium NO_2_^−^ (LM)	43	262	0.51	3.79
Low NO_3_^−^, low NO_2_^−^ (PL)	8	5.8	0.09	0.08

Nitrate (NO_3_^−^); Nitrite (NO_2_^−^).

**Table 2:** Baseline characteristics of participants, Mean ± SD.

Participant Characteristics	Total (*n* = 11)
Age (y)	24 ± 5.7
Height (cm)	173 ± 8.9
Body mass (kg)	67.6 ± 13.3
Plasma NO_2_^−^ (μM)	0.77 ± 0.086 *
Plasma NO_3_^−^ (μM)	36.74 ± 1.89 *
SBP (mmHg)	111.36 ± 1.73 *
DBP (mmHg)	70.83 ± 1.22 *

Systolic Blood Pressure (SBP); Diastolic Blood Pressure (DBP). * Values are taken as a mean of eligible participants measured at 0 h for each trial.

## Data Availability

The data presented in this study are available on request from the corresponding author.
